# A Cross-Sectional Study of the Perception of Individualized Nursing Care Among Nurses in Acute Medical and Perioperative Settings

**DOI:** 10.3390/nursrep14040232

**Published:** 2024-10-25

**Authors:** Ana Ramos, Sara Pires, Eunice Sá, Idalina Gomes, Elisabete Alves, César Fonseca, Anabela Coelho

**Affiliations:** 1 Nursing Research Innovation and Development Centre of Lisbon (CIDNUR), Nursing School of Lisbon (ESEL), 1600-190 Lisboa, Portugal; sarapires@esel.pt (S.P.); esa@esel.pt (E.S.); idgomes@esel.pt (I.G.); 2 Comprehensive Health Research Center (CHRC), University of Évora, 7004-516 Évora, Portugal; elisabete.alves@uevora.pt (E.A.); cfonseca@uevora.pt (C.F.); anabela.coelho@uevora.pt (A.C.); 3 Global Health and Tropical Medicine, Instituto de Higiene e Medicina Tropical, Universidade NOVA de Lisboa, 1099-085 Lisboa, Portugal

**Keywords:** patient individualization, nursing care, acute care, perioperative care

## Abstract

**Background/Objectives:** Individualized nursing care allows for systematic assessment and intervention; considers a patient’s preferences, values, and context; and contributes to a positive care trajectory. However, its operationalization has proven to be challenging. This research aimed to evaluate nurses’ perceptions of individualized care and analyze their relationship with sociodemographic variables. **Methods:** A cross-sectional study was conducted on 122 eligible and registered nurses at a Hospital Center, in the Ophthalmology (operating room and inpatient ward) service, the Cardiology service, the Internal Medicine service, and the Medical Emergency Unit, for adults/older adults in Portugal. The nursing version of the Individualized Care Scale (ICS-Nurse) was used for the assessment, including three sub-dimensions: clinical situation, personal life situation, and decisional control over care-related decisions. Cronbach’s alpha and principal component analysis were used for the data analysis. The STROBE checklist was used to report this study. **Results:** No statistically significant differences were found based on the age, gender, level of education, or years of professional experience of the nurses within the sub-dimensions of individualization. The nurses overall had a good perception of the importance of individualized care (4.06 ± 0.46 ICS-A-NURSE) but faced difficulties in its implementation during the last shift they worked (3.97 ± 0.49 ICS-B-NURSE). **Conclusions:** The items considered to be of greatest importance were the response to the physical and emotional needs arising from illness and assistance in decision-making through educational instructions. Aspects related to the personal lives of patients, such as family inclusion in an individual’s care plan, everyday habits, and previous experiences of hospitalization, received the lowest scores. Recognizing priority areas for improvement in the individualization of nursing care can contribute to developing training programs and policies that promote a holistic approach. Future studies should consider patient outcomes related to their needs for individualization.

## 1. Introduction

The global population is experiencing a significant demographic shift, with an increased number of older adults. This trend, known as demographic aging, is driven by declining birth rates and increased life expectancy. According to the World Health Organization [1], in 2030, 1 in 6 people worldwide will be 60 years old or older. At that time, the proportion of the population aged 60 and over will increase from 1 billion in 2020 to 1.4 billion. By 2050, the global population of 60 and over to will double to 2.1 billion. The number of people aged 80 and over is projected to triple between 2020 and 2050, reaching 426 million. Portugal is one of the countries with the highest Aging Index in the world, and recent statistics place Portugal as the fourth fastest aging country [2]. With increased longevity, older adults have had a higher dependency rate, rising from 24.4% in 2001 to 37.6% in 2022 [3]. Storeng et al. [4] concluded in a cross-sectional analysis that individuals aged 60 to 69 who suffer from three or more diseases fit within a profile of complex morbidity. These individuals are those who develop severe disability in performing basic activities of daily living over the years and have a moderate risk of mortality. Aging leads to a progressive reduction in functional reserve mechanisms and homeostatic capacities, and factors such as financial insecurity, loneliness, and psychosocial concerns, such as fear of the future and death, further intensify the vulnerability of older adults [5]. Advanced age (≥85 years), no school attendance, a low body mass index, and the presence of a sad or depressed mood are predictive variables for a deficit in functional capacity, where hospitalization can aggravate vulnerability. Once disability sets in, its rehabilitation is slow and difficult, but individualized nursing care can contribute to preventing dependency in older adults [6]. Standardized protocols or treatments in a hospital setting are not synonymous with better outcomes [7].

In perioperative and acute care settings, understanding illness and planning surgery represent situational transitions of health and illness for both a person and their family, which involve uncertainties, fragility, and risk to life [8]. Exacerbation of chronic illness or a sudden event can affect a person’s health condition, potentially leading to functional deficits (loss or alteration of an anatomical, physiological, or psychological structure or function), activity restrictions (limitation or loss of the ability to perform usual activities due to functional deficits), and limitations in participation in decision-making about their health project [6,8,9]. Therefore, the relationship between a nurse, a hospitalized person, and their family needs to be supported by a partnership intervention model. This model should ensure genuine sharing of power and the right to make choices [10]. The implementation of safe, individualized, and quality care is a universal concern [11], so it is necessary to innovate healthcare to effectively respond to increasingly complex therapeutic regimens [12]. Individualized nursing care is essential for addressing the complex and diverse needs of patients in various healthcare settings. It goes beyond the one-size-fits-all model, recognizing that each patient’s health status, personal history, and preferences play a crucial role in shaping their care needs [13,14,15,16,17]. Individualized nursing care can be defined as a set of interventions that consider patients’ characteristics, clinical conditions, personal life situations, and preferences to promote active patient participation in decision-making [13,14]. Delivering individualized nursing care offers a multidimensional approach to patient assessment, addressing individuals’ physical, emotional, social, and spiritual needs [17]. This involves considering and incorporating aspects of gender, religion, ethnicity, and ideology into care while addressing pathophysiological, psychological, mental, and socio-economic conditions. Individualized nursing care aligns with the principles of patient-centered care, a model increasingly recognized as essential for improving healthcare outcomes by focusing on the specific needs, preferences, and circumstances of each patient [15,16,17].

For individualized nursing care, establishing an interpersonal, ethical, and understanding relationship is essential, serving as the key to successfully identifying and implementing the most appropriate nursing intervention [18]. This relationship enables understanding an individual and their context, as well as fostering a collaborative approach with the patient or caregiver to promote self-care in its dual sense: self-care of oneself and others [10]. So, the definition of individualized care includes the various actions that occur during interactions between nurses and patients. Initially, nurses obtain wide information regarding a patient’s preferences, needs, fears, and viewpoints. Next, they mobilize these data about the patient’s traits and circumstances, as well as their responses to health issues, to plan and execute the necessary activities and interventions. Lastly, nurses encourage patient involvement in the planning and implementation of nursing interventions [19,20]. From the patients’ perspective, individualized care should be defined based on their evaluation, perception, or understanding of the interventions provided by nurses [16]. From the nurses’ perspective, individualized care is achieved when interventions are adjusted to meet and respond to the specific needs of each patient [20,21].

Recent studies have demonstrated the positive effect of individualized care on patient outcomes. For instance, patients with chronic obstructive pulmonary disease receiving individualized nursing care had better results in longer walking distances, in all domains of both the physical and mental composites, and improved quality of life compared with patients receiving traditional nursing care [22]. According to Suhonen et al. [17], individualized interventions impact the effectiveness of educational interventions, rehabilitation success, satisfaction with nursing care, quality of life improvements, individual autonomy, the cost-effectiveness of nursing interventions, the quality of communication, adherence to therapeutic regimens, and increased motivation and job satisfaction among nursing teams. Individualized nursing was identified as a feasible intervention that resulted in rehabilitation after percutaneous coronary procedures, with an improvement in the levels of anxiety, sleep quality, and quality of life, as well as a reduction in postoperative complications [23].

The evaluation of individualized nursing care with the Individualized Care Scale (ICS) is an approach to measuring the perception and implementation of care adapted to the needs and wishes of patients. The ICS allows for a better understanding of how nursing interventions can be adjusted to improve the quality of care provided and achieve better patient outcomes [16]. Previous studies have assessed nurses’ perceptions of individualized care in medical and surgical wards [12,16,20,21,22,23], intensive care units [13], and primary care settings [20]. This study integrates new contexts, such as emergency and perioperative care, specifically in the operating room. Despite its benefits, the implementation of individualized nursing care faces several challenges. These include limitations in resources and variability in care practices, organizational culture, leadership styles, and ratios [24,25]. Nonattendance to individualized care results in insufficient identification of patient dimensions, which have an important role in unmet needs that can compromise the health trajectories of people, families, and caregivers [26,27,28]. Therefore, this research was conducted to evaluate the individualized care perceptions of nurses in acute medical and perioperative settings. Two hypotheses were formulated: (1) Do nurses have a positive perception of the level of individualization of care implemented in clinical practice? (2) Are the sociodemographic variables of nurses (age, gender, years of education, and years of professional experience) associated with their perception of individualized care?

## 2. Materials and Methods

### 2.1. Research Aim, Design, and Setting

This study aimed to characterize the sociodemographic profile of nurses in acute medical and perioperative care settings and identify the items of nursing care individualization, through ICS-A-NURSE and ICS-B-NURSE, that nurses perceive as most integrated into their clinical practice. This was a cross-sectional study including two assessments, ICS-A-NURSE and ICS-B-NURSE, which are primary tools utilized to measure nurses’ perceptions regarding the customization of care including the nursing version of the Individualized Care Scale (ICS-Nurse), which is better suited to acute care environments. The two scales were applied simultaneously from 10 January to 29 February 2024 in Portugal (Lisbon) at a University Hospital Center, in the Ophthalmology Service (operating room and inpatient ward), in the Cardiology Service, in the Medicine Service, and in the Medical Emergency Unit. Each unit manages different patient conditions, ranging from acute to chronic illnesses. This diversity provides a rich context for evaluating how nurses perceive and practice individualized care across various clinical scenarios, enhancing this study’s comprehensiveness. The nursing staff in these units were readily available and willing to participate in the survey, ensuring a good sample size. Their involvement was crucial to accurately capturing perceptions regarding individualized nursing care.

This research adhered to the STROBE guidelines for cross-sectional studies [29] and is part of Study I of the Research Project titled “Individualization of Care: The Nursing Partnership Intervention in Hospital Settings” (INbyCARE). It aims to establish the baseline of nurses’ perceived and provided individualized care, which will contribute to the characterization of usual care and stratify the aspects of individualization that are less incorporated into clinical practice. This will enable the subsequent implementation of a Partnership Intervention Program [10] designed to enhance the implementation of individualized care in the environments included.

### 2.2. Participants and Measures

The sample comprised 112 nurses practicing in acute medical and perioperative settings, of 146 nurses (participation rate = 76.7%). To minimize selection bias, efforts were made to ensure that the sample of nurses included in this study was representative of the population studied. The sample was non-probabilistic, based on convenience [30]. The inclusion criteria were (i) nurses with at least 6 months of professional experience in the medical and surgical specialty services where they are allocated; (ii) nurses who expressed voluntary and informed consent to participate in the study. Nurse managers were also invited to participate, as the literature suggests that different leadership styles can either facilitate or inhibit the process of individualization [22,23].

ICS-Nurse is a self-administered scale developed originally in Finland by Suhonen et al. [16]. Amaral et al. [21] translated and validated an ICS-Nurse (A and B) scale for the Portuguese population. An email was sent to the author [21], who responded by granting permission for the use of the translated scale. The ICS-A-Nurse subscale aims to assess nurses’ perceptions of how they support their patients’ individuality through specific nursing activities during their current activity. The ICS-B-Nurse subscale seeks to assess nurses’ perceptions of how they evaluate the maintenance of individuality in their care, such as during their last shift. Within these two dimensions, individualized care includes three subscales: clinical situation (ClinA-Nurse and ClinB-Nurse) (items: 1–7), personal life situation (PersA-Nurse and PersB-Nurse) (items: 8–11), and decisional control over care-related decisions (DecA-Nurse and DecB-Nurse) (items: 12–17). The response options range from 1 to 5 (1 = strongly disagree; 2 = disagree to some extent; 3 = neither agree nor disagree; 4 = agree to some extent; 5 = strongly agree). Higher scores indicate a higher perception of individuality in care [20].

The clinical situation sub-dimension explored care behaviors that assist in maintaining patients’ individuality concerning their reactions to illness, their emotions, and the personal significance of an illness. The personal life situation sub-dimension examines care behaviors that help preserve patients’ individuality concerning their beliefs and values, routines, activities, preferences, family relationships, and experiences both at work and in the hospital. The decisional control over care sub-dimension investigates care behaviors that support patients’ individuality by considering their emotions, thinking, and requests and their ability to have input and participate in decisions regarding their care [16,17]. Potential confounding factors, such as group age, gender, level of education, and years of professional experience, were measured and accounted for during the analysis.

### 2.3. Research Ethics

This study’s protocol was submitted and reviewed by the Health Ethics Committee of the University Hospital Center, where the research was conducted, and received approval number I/25010/2023 (16 November 2023). All participants were provided with comprehensive information about this study’s objectives, procedures, potential risks, and benefits. Written informed consent was obtained from all participants before their inclusion in this study, ensuring they understood their rights, including their right to withdraw from the study at any time without any consequences.

Data were collected via responses to a provided link and securely stored with password protection. Access to the data was restricted exclusively to the principal investigators, ensuring confidentiality and data integrity.

### 2.4. Data Analysis

Statistical analysis was conducted using IBM SPSS Statistics 27 software. A descriptive analysis was performed to examine the sociodemographic and employment characteristic variables. No missing data were encountered in this study. All participants completed the required questionnaires in full, ensuring a complete dataset for analysis. The use of a structured and standardized data collection process, combined with close monitoring during data collection, contributed to the completeness of the data.

Internal consistency reliability analysis was assessed using Cronbach’s alpha to ensure that the items on the scale reliably measured the dimensions of individualized nursing care. Cronbach’s alpha values above 0.7 are generally considered acceptable, with values closer to 1 indicating better internal consistency [30,31]. Principal component analysis (PCA) was used on a validated scale to understand the relationships between the variables better, with the Kaiser–Meyer–Olkin index confirming the adequacy of the data for this analysis. The Kolmogorov–Smirnov (K-S) test showed that the variables related to the individualization of nursing care did not follow a normal or uniform distribution in the population. The Kruskal–Wallis test was considered as a non-parametric alternative to one-way ANOVA [32]. However, no statistically significant differences were found between the relationship of age groups, education level, and professional experience and the sub-dimensions of the individualization of care. In all statistical tests, only a 5% acceptable error probability was considered, meaning that a result was deemed statistically significant if *p* < 0.05 [30].

## 3. Results

### 3.1. Characteristics of the Participants

The sample consisted of 112 nurses, of which 14.3% were from the Ophthalmology Service, 26.8% from the Medical Service, 24.1% from the Cardiology Service, and 34.8% from the Medical Emergency Unit (Table 1). The average age of the nurses included was 39.4 years ± 12.736 (minimum age = 22 years and maximum age = 65 years), with the most represented age group being 36 to 40 years. Regarding gender, 82.1% were female, and 72.3% of the total nurses held an undergraduate degree, followed by a master’s degree (14.3%) and a postgraduate degree (13.4%). Concerning the professional category, 75.0% were nurses; 24.1% were specialist nurses; and 1.0% were nurse managers. Most of the nurses had 11 or more years of professional experience (59.8%).

### 3.2. Sub-Dimensions and Items of Individualization Nursing Care

The principal component analysis (PCA) revealed key findings for the sub-dimensions of individualized nursing care. For the sub-dimension of clinical situation, the Cronbach’s alpha values were 0.862 for ICS-A-NURSE and 0.868 for ICS-B-NURSE, indicating excellent internal consistency (Table 2 and Table 3). The Kaiser–Meyer–Olkin (KMO) values were 0.867 for ICS-A-NURSE and 0.877 for ICS-B-NURSE, suggesting an excellent correlation between the variables.

For the personal life situation sub-dimension, the Cronbach’s alpha values were 0.748 for ICS-A-NURSE and 0.759 for ICS-B-NURSE, indicating good internal consistency. The KMO values were 0.697 for ICS-A-NURSE and 0.736 for ICS-B-NURSE, showing a moderated correlation between the variables.

The decisional control sub-dimension presented varied Cronbach’s alpha values of 0.684 for ICS-A-NURSE and 0.774 for ICS-B-NURSE (acceptable). The KMO values were 0.719 for ICS-A-NURSE and 0.829 for ICS-B-NURSE, indicating good adequacy, especially for ICS-B-NURSE.

These results indicate that the internal consistency of the indicators of the sub-dimensions was more robust for clinical situation and decisional control (ICS-B-NURSE) than for personal life situation and decisional control (ICS-A-NURSE). This suggests that nurses’ perceptions of the individualization of care are more cohesive and well defined in the dimensions related to clinical situations and decisional control than in the personal life situations of patients.

**Figure 1:**
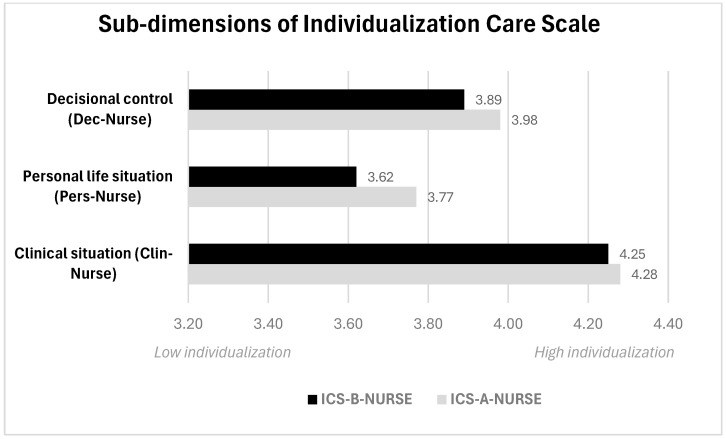
Summary of the sub-dimensions of ICS-Nurse.

These results indicate that the ICS-A-NURSE group consistently reported higher levels of individualization across all three sub-dimensions compared to the ICS-B-NURSE group (Figure 1). These results suggest that while the nurses valued the sub-dimensions of individualization in overall care, their incorporation into their most recent nursing shifts was lower. Higher scores were obtained in the clinical situation sub-dimension (ICS-A-NURSE = 4.28 and ICS-B-NURSE = 4.25), while lower scores were obtained in the inclusion of aspects that incorporated the personal life situation dimension (ICS-A-NURSE = 3.77 and ICS-B-NURSE = 3.62).

The highest scoring items included “Instructions to patients” (ICS-A: 4.62; ICS-B: 4.37); “Needs that require care and attention” (ICS-A: 4.35; ICS-B 4.44); and “Feelings about illness/health condition” (ICS-A: 4.38; ICS-B 4.31), as shown in Table 4.

Both responses to the scales had lower averages in items such as “Ask patients at what time they want to wash” (ICS-A: 3.21; ICS-B: 3.11); “Family to take part in their care” (ICS-A: 3.46; ICS-B: 3.55); and “Previous experiences of hospitalization” (ICS-A: 3.57; ICS-B 3.52), indicating areas for potential improvement in the individualization of nursing care. There is a need too for greater focus on patient participation in decisions (ICS-A: 3.93; ICS-B: 3.76) and understanding patients’ daily habits (ICS-A: 3.09; ICS-B: 3.66).

The items that they considered at an intermediate level were encouraging patients to express their opinions; giving them a chance to take responsibility as far as possible; understanding what they want to know about their illness or health condition; considering patients’ wishes regarding their care; exploring what the illness condition means to them; and talking with patients about their fears and anxieties.

## 4. Discussion

The sociodemographic characteristics of nurses have been associated with the degree of individualization of the care they provide; however, in this study, no statistically significant differences were found. Nurses with more professional experience, advanced nursing education, or postgraduate qualifications tend to provide more individualized care [33]. Nurses with more years of experience and higher education levels possess the skills and maturity needed to deliver tailored patient care effectively [34]. Previous investigations have confirmed that higher levels of education, namely a master’s degree, among nurses correlate with greater decision-making autonomy, which is crucial for integrating individualized care into their practice [35,36].

The Cronbach’s alpha coefficients were reported as 0.88 (range: 0.72–0.83) for the ICS-A-Nurse subscales and 0.90 (range: 0.73–0.84) for the ICS-B-Nurse subscales, with the overall Cronbach’s alpha coefficient being 0.88 in the study by Suhonen et al. [16] and 0.91 in the research conducted by Acaroglu et al. [37]. In a cross-cultural international study, the alpha coefficients for ICS-A and ICS-B were 0.88 and 0.87 in Finland; 0.95 and 0.84 in Greece; 0.91 and 0.90 in Portugal; and 0.95 and 0.93 in the U.S.A., respectively [38]. In this study, the Cronbach’s alpha values were 0.886 for ICS-A-NURSE and 0.905 for ICS-B-NURSE. These values are consistent with those found in previous studies, suggesting that the scales used are reliable and internally consistent, additionally confirmed by the PCA, with all sub-dimensions having a KMO value > 0.697 (*p* < 0.001). The high values of Cronbach’s alpha in all of these studies demonstrate that the items within each subscale and the overall scales measure the same underlying concept effectively.

The average score of 4.06 ± 0.46 obtained by the nurses from ICS-A-NURSE indicates a good perception of individualized care among the participants. ICS-B-NURSE presented a slightly lower score of 3.97 ± 0.49. Comparing these findings with those of other studies conducted in the Netherlands and Belgium, the nurses’ mean scores obtained from ICS-A-NURSE were higher (4.23 ± 0.58) [39]. Turkish nurses’ mean score obtained from ICSA-Nurse was 3.96 ± 0.72 [36]. Another study conducted in Turkey of nurses working in intensive care, surgery, and internal medicine services at a state hospital, with a sample of 97 nurses, found that the mean score of the total items in ICS-A for the nurses was 3.75 ± 0.74 [40]. The observed differences in the average scores may be attributed to the typical four-year duration of nursing education in Portugal, coupled with the country’s distinct healthcare culture, both of which influence the levels of individualized nursing care. The duration and cultural context of nursing education can vary significantly across different countries, potentially impacting how nurses approach patient care [34]. This variability may result in differing emphases on patient-centered practices, highlighting the need for a tailored approach to nursing education that considers cultural and contextual factors in various healthcare settings.

ICS-A-NURSE demonstrated higher average scores across most of the items evaluated, which denotes an overall recognition by nurses of the need for individualized care, but difficulties in its translation into practice were evident in ICS-B-NURSE, which provides important insights. This study found a greater emphasis on the sub-dimension of clinical situation (ClinA and ClinB), which reflects concern with integrating the current state of illness; how it affects a patient’s physical, psychological, and emotional well-being; and what needs require intervention [41,42]. In contrast, other research places greater weight on decisional control (DecA), which facilitates patients making their own choices about their care [43,44].

Feelings about health conditions received notably high scores, indicating a strong prominence of clear communication, attentiveness to patient needs, and emotional support. Babaei et al. [45] found that emotional support and understanding patients’ feelings about their illness are essential components of high-quality nursing care. Their study revealed that nurses who actively engage in empathetic communication and provide emotional support can significantly reduce patient anxiety and improve overall patient satisfaction. This approach not only helps in building trust and rapport between nurses and patients but also contributes to better adherence to treatment plans and faster recovery. This study also emphasized the importance of training nurses in emotional intelligence and communication skills to enhance their ability to deliver individualized care effectively.

The instructions provided to patients were a highly valued item, included in the sub-dimension of decisional capacity, which may be particularly associated with the promotion of self-care and self-management. This is a very relevant aspect of individualization in the control of multimorbidity, mainly in diabetes mellitus [46]. The relatively low scores on asking patients about their preferred times for personal care routines like washing can reflect a lack of personalization in care schedules. This omission can lead to decreased patient satisfaction and a sense of autonomy, which are vital for recovery and overall well-being. A low level of individualized care results in insufficient identification of a person’s needs, contributing to missed nursing care [47]. High rates of missed nursing care have been documented, ranging from 52% to 86% [48,49]. Ergezen et al. [50]’s research showed that emotional support, patient bathing, and ambulation are the most often overlooked nursing care activities. Nurses should recognize a person as responsible for their own life and health project, endowed with the power to make decisions and with the right to participate in their care [10]. The dimension of a person’s personal life allows both relevant objective information (such as personal and surgical history, current illness history, usual clinical reference values, level of functional capacity, and formal and informal care networks) and significant subjective information (including daily living activities, instrumental activities, occupation/recreation, life goals, and values related to social and cultural identity) to be captured. The collection and utilization of information regarding personal life enhance empathy and understanding, which can, in turn, promote positive care relationships essential for fostering independence in self-care. Information about personal life can serve as an important asset for health policies focused on integrating care, as it provides the opportunity to create a database of clinical information that captures how a person and their care needs change throughout life. This perspective allows for anticipating the allocation of health resources and the needs of nursing care [51].

Incorporating family into patient care had one of the worst scores. Family involvement is a key component of patient-centered care, contributing to better management of chronic conditions, improving patient compliance, and enhancing emotional support [52]. The COVID-19 pandemic significantly impacted the establishment of interpersonal relationships between nurses and patients, primarily due to the use of personal protective equipment and the fear of contamination. Additionally, restrictions imposed to limit the spread of the virus restricted family access to healthcare units, creating a critical barrier to the integration of families into patient care. This situation not only isolated patients but also diminished nurses’ ability to involve family members in the care process, potentially contributing to a regression in the individualization of care and hindering the promotion of a more holistic and collaborative care environment [53]. Wong et al. [54] developed and evaluated a multidisciplinary intervention for patients undergoing surgery, which included patient education and family integration into care, and concluded that it improved health literacy for both. However, when nurses do not adequately involve families, it can lead to increased stress for the patient and a potential decline in the quality of care, as verified in perioperative and critical care [55,56]. Kiston et al. [57] argue that a care biography is vital to nursing fundamental care. It guarantees life and well-being because it considers a personalized account of an individual’s self-care abilities, capacity for care, family relationships, and understanding of the care they receive and expect from others. Like a personalized health record, a care biography tracks a person’s self-care skills, preferences, and needs throughout their life, as confirmed by Ramos et al. [58] and Fonseca et al. [59]. Fundamental care encompasses the essential support required for everyone to ensure survival, maintenance, protection, or a peaceful end, regardless of their clinical condition or care environment [60]. When the provision of fundamental care is inadequate, it negatively affects patients, families or caregivers, healthcare professionals, and the overall healthcare system [49,61]. It also contributes to the risk of adverse events when the planned or recommended care is not implemented [49].

## 5. Limitations of This Study

This study’s cross-sectional nature provides only a view of individualized nursing care at one point in time, limiting its ability to assess changes or trends over time. Its sample is not representative of all acute medical and perioperative settings because it was non-probabilistic, which does not allow for generalization. Furthermore, the sample did not follow a normal distribution, so the use of parametric statistical tests was not possible, and it would have been more robust if it had. This study may not have adequately controlled for potential confounding factors that could have affected nurses’ perceptions of individualized care—for example, the workload related to the time of year at which the data were collected, which corresponds to winter in Portugal, a peak season for respiratory infections that strains the National Health System, as well as previous experience with individualized care, leadership style, or the prevailing organizational culture. Another significant limitation of this study is its cross-sectional design, which inherently restricts the ability to establish cause-and-effect relationships or analyze changes in behavior over time. In collecting data at a single point, this study cannot determine how perceptions of individualized nursing care may evolve or how they might influence patient outcomes.

## 6. Conclusions

Future efforts should focus on addressing the gaps identified to improve individualized care. This includes adopting more flexible scheduling practices to accommodate patient preferences, enhancing family involvement in care processes, and fostering a more participatory approach to decision-making. The aspects most integrated and valued by nurses in acute medical and perioperative care were instructions to patients, needs that require care and attention, feelings about illness/health conditions, and how their health condition affects them, highlighting an emphasis on the physical and psycho-emotional dimensions. No statistically significant differences were found between the sociodemographic characteristics of the nurses and their perceptions of individualized care.

Furthermore, continuous professional development and training programs emphasizing these aspects can further strengthen the implementation of individualized care practices. Integrating these improvements can lead to better patient outcomes, reduced missed care, increased satisfaction, and overall improved quality of care. It is essential to highlight the critical role of nursing education in preparing future nurses for individualized care of patients. Well-structured knowledge and learning experiences in this area are vital for equipping nurses with the skills necessary to assess and respond to the diverse needs of their patients. Emphasizing individualized care in nursing curricula enhances the quality of patient care and promotes better health outcomes. So, educational programs focused on effective communication, cultural competence, and family involvement should be created to ensure that nurses are adept at delivering personalized interventions that truly address the complexities of each patient’s situation. Integrating technology, such as clinical decision support systems, wearable devices, mobile health applications, and telehealth, can also facilitate collecting and responding to comprehensive patient data, enhancing nurses’ ability to give personalize care based on individual histories and preferences. Policies promoting interprofessional collaboration and integrated care planning should also be encouraged to ensure that all healthcare providers involved in a patient’s care align with their approach to individualized care.

We suggest conducting future studies that incorporate the perspectives of patients into acute and perioperative care for comparative analyses with the views of nurses. The integration of qualitative study designs is recommended to identify the factors that influence the individualization of nursing care most significantly. Additionally, pilot studies should be undertaken to demonstrate the effectiveness of individualized care in populations with health conditions.

## Figures and Tables

**Table 1:** Sociodemographic characteristics of nurses in acute medical and perioperative settings.

Setting of Care	Ophthalmology	Medicine	Cardiology	Medical Emergency Unit	Total
	N (%)	N (%)	N (%)	N (%)	N (%)
Age category (years)					
≤25	0 (0.0)	2 (1.8)	5 (4.5)	9 (8.0)	16 (14.3)
26–30	0 (0.0)	8 (7.1)	5 (4.5)	8 (7.1)	21 (18.8)
31–35	1 (0.9)	3 (2.7)	3 (2.7)	1 (0.9)	8 (7.1)
36–40	5 (4.5)	5 (4.5)	5 (4.5)	9 (8.0)	24 (21.4)
41–50	3 (2.7)	8 (7.1)	3 (2.7)	6 (5.4)	20 (17.9)
≥51	7 (6.3)	4 (3.6)	6 (5.4)	6 (5.4)	23 (20.5)
Gender					
Female	12 (12.5)	26 (23.2)	20 (17.9)	32 (28.6)	92 (82.1)
Male	2 (1.8)	4 (3.6)	7 (6.3)	7 (6.3)	20 (17.9)
Level of education					
Undergraduate degree	12 (10.7)	22 (19.6)	20 (17.9)	27 (24.1)	81 (72.3)
Postgraduate	2 (1.8)	7 (6.3)	2 (1.8)	4 (3.6)	15 (13.4)
Master’s	2 (1.8)	1 (0.9)	5 (4.5)	8 (7.1)	16 (14.3)
Professional category					
Nurse	13 (11.6)	21 (18.8)	21 (18.8)	29 (25.9)	84 (75.0)
Specialized nurse	3 (2.7)	9 (8.0)	6 (5.4)	9 (8.0)	27 (24.1)
Nurse manager	0 (0.0)	0 (0.0)	0 (0.0)	1 (0.9)	1 (0.9)
Professional experience (years)					
≤2	0 (0.0)	3 (2.7)	5 (4.5)	10 (8.9)	18 (16.1)
3–5	0 (0.0)	6 (5.4)	3 (2.7)	4 (3.6)	13 (11.6)
6–10	1 (0.9)	3 (2.7)	5 (4.5)	5 (4.5)	14 (12.5)
≥11	15 (13.4)	18 (16.1)	14 (12.5)	20 (17.9)	67 (59.8)
Total	16 (14.3)	30 (26.8)	27 (24.1)	39 (34.8)	112 (100.0)

**Table 2:** Analysis of the main components of ICS-A-NURSE and alpha coefficients (N = 112).

Items Content	Component Matrix			h^2^
ICS-A-NURSE	Clinical Situation (Clin-Nurse)			
Feelings about illness/health condition	0.827			0.706
Needs that require care and attention	0.842			0.563
Chance to take responsibility as far as possible	0.857			0.445
Identify changes in how they have felt	0.847			0.506
Talk with patients about fears and anxieties	0.835			0.628
Find out how their health condition affects them	0.844			0.528
What the illness/health condition means to them	0.847			0.522
	Personal life situation (Person-Nurse)			
What kind of things they do in their everyday life		0.689		0.606
Previous experiences of hospitalization		0.733		0.475
Everyday habits		0.592		0.774
Family to take part in their care		0.735		0.464
	Decisional control (Dec-Nurse)			
Instructions to patients			0.687	0.617
What they want to know about illness/health condition			0.669	0.559
Patients’ wishes regarding their care			0.619	0.598
Help patients take part in decisions			0.613	0.671
Encourage patients to express their opinions			0.592	0.585
Ask patients at what time they want to wash			0.671	0.530
Cronbach’s alpha	0.862	0.748	0.684	0.886
Kaiser–Meyer–Olkin	0.867	0.697	0.719	
Bartlett’s test of sphericity	321.380	118.334	122.062	
	21	6	15	
	<0.001	<0.001	<0.001	

**Table 3:** Analysis of the main components of ICS-B-NURSE and alpha coefficients (N= 112).

Itens Content	Component Matrix			h^2^
ICS-B-NURSE	Clinical situation (Clin-Nurse)			
Feelings about illness/health condition	0.842			0.635
Needs that require care and attention	0.844			0.627
Chance to take responsibility as far as possible	0.898			0.241
Identify changes in how they have felt	0.837			0.681
Talk with patients about fears and anxieties	0.840			0.682
Find out how their health condition affects them	0.843			0.647
What the illness/health condition means to them	0.841			0.654
	Personal life situation (Person-Nurse)			
What kind of things they do in their everyday life		0.638		0.736
Previous experiences of hospitalization		0.663		0.695
Everyday habits		0.687		0.626
Family to take part in their care		0.806		0.320
	Decisional control (Dec-Nurse)			
Instructions to patients			0.744	0.485
What they want to know about illness/health condition			0.730	0.538
Patients’ wishes regarding their care			0.729	0.573
Help patients take part in decisions			0.701	0.668
Encourage patients to express their opinions			0.711	0.633
Ask patients at what time they want to wash			0.824	0.177
Cronbach’s alpha	0.868	0.759	0.774	0.905
Kaiser–Meyer–Olkin	0.877	0.736	0.829	
Bartlett’s test of sphericity	392.166	128.400	199.846	
	21	6	15	
	<0.001	<0.001	<0.001	

**Table 4:** Description of ICS-Nurse items.

Items	ICS-A-NURSE			ICS-B-NURSE		
	Mean ± SD	Median	Range	Mean ± SD	Median	Range
Clinical situation (Clin-Nurse)						
Feelings about illness/health condition	4.38 ± 0.602	4	2–5	4.31 ± 0.658	4	2–5
Needs that require care and attention	4.45 ± 0.551	4	3–5	4.44 ± 0.582	4	3–5
Chance to take responsibility as far as possible	4.19 ± 0.704	4	2–5	3.95 ± 0.909	4	1–5
Identify changes in how they have felt	4.23 ± 0.585	4	3–5	4.29 ± 0.653	4	3–5
Talk with patients about fears and anxieties	4.32 ± 0.647	4	2–5	4.24 ± 0.661	4	3–5
Find out how their health condition affects them	4.36 ± 0.656	4	2–5	4.29 ± 0.653	4	3–5
What the illness/health condition means to them	4.05 ± 0.733	4	2–5	4.21 ± 0.659	4	3–5
Personal life situation (Person-Nurse)						
What kind of things they do in their everyday life	4.16 ± 0.789	4	2–5	3.73 ± 0.939	4	2–5
Previous experiences of hospitalisation	3.57 ± 0.984	4	1–5	3.52 ± 0.986	4	1–5
Everyday habits	3.90 ± 0.920	4	2–5	3.66 ± 0.876	4	1–5
Family to take part in their care	3.46 ± 0.958	4	1–5	3.55 ± 1.02	4	1–5
Decisional control (Dec-Nurse)						
Instructions to patients	4.62 ± 0.524	5	3–5	4.37 ± 0.615	4	3–5
What they want to know about illness/health condition	4.02 ± 0.849	4	2–5	4.06 ± 0.763	4	1–5
Patients’ wishes regarding their care	4.17 ± 0.599	4	3–5	4.11 ± 0.662	4	2–5
Help patients take part in decisions	3.93 ± 0.791	4	2–5	3.76 ± 0.713	4	2–5
Encourage patients to express their opinions	3.93 ± 0.824	4	2–5	3.94 ± 0.675	4	2–5
Ask patients at what time they want to wash	3.21 ± 1.08	3	1–5	3.11 ± 1.02	3	1–5

## Data Availability

Data are available from the authors upon reasonable request.

## Public Involvement Statement

No public involvement in any aspect of this research.

## Guidelines and Standards Statement

This manuscript was drafted against the Strengthening the Reporting of Observational Studies in Epidemiology (STROBE) Statement: guidelines for reporting cross-section studies.
